# Supplementary material for: Electron Attachment Studies with the Potential Radiosensitizer 2-Nitrofuran
Source: Int J Mol Sci. 2020 Nov 24;21(23):8906. doi: 10.3390/ijms21238906 (PMC7727711; doi:10.3390/ijms21238906)
Supplement: Supplementary file 1 [file ijms-21-08906-s001.pdf]

Supplementary Information for

Dissociative Electron Attachment Studies with 2-Nitrofuran

Muhammad Saqib[1,2], Eugene Arthur-Baidoo[1,2], Milan Ončák[1*], and Stephan Denifl[1,2*]

[1]Institute for Ion Physics and Applied Physics, University of Innsbruck, Technikerstrasse 25, 6020 Innsbruck, Austria

[2]Center for Biomolecular Sciences Innsbruck, University of Innsbruck, Technikerstrasse 25, A-6020 Innsbruck, Austria

* corresponding authors: milan.oncak@uibk.ac.at, stephan.denifl@uibk.ac.at

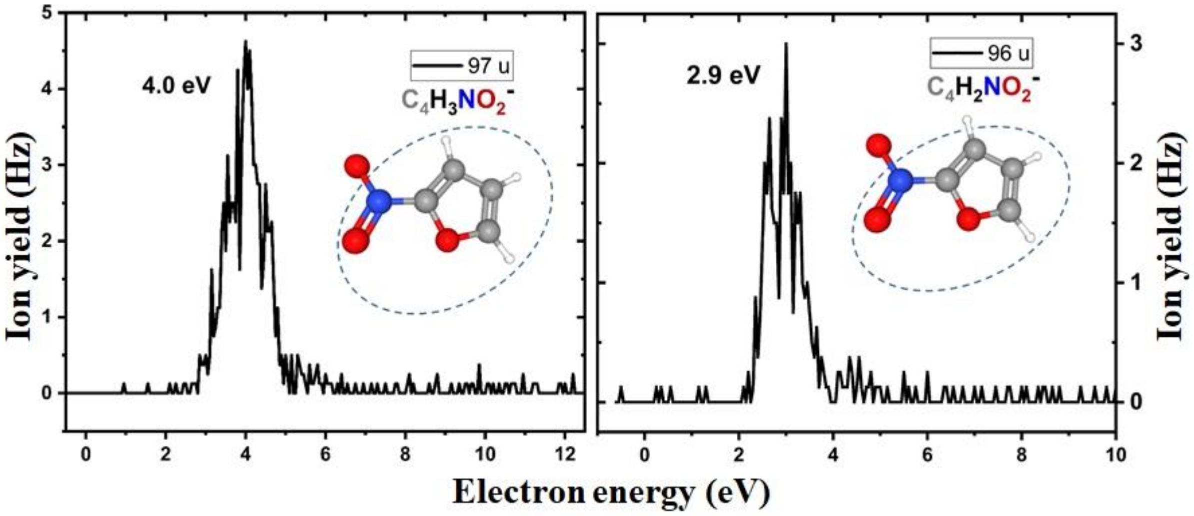

Figure S1: Anion efficiency curves for the anionic fragments (2-NIF – O)⁻ and (2-NIF – OH)⁻ with masses 97 u and 96 u, formed upon DEA to 2-NIF, respectively.

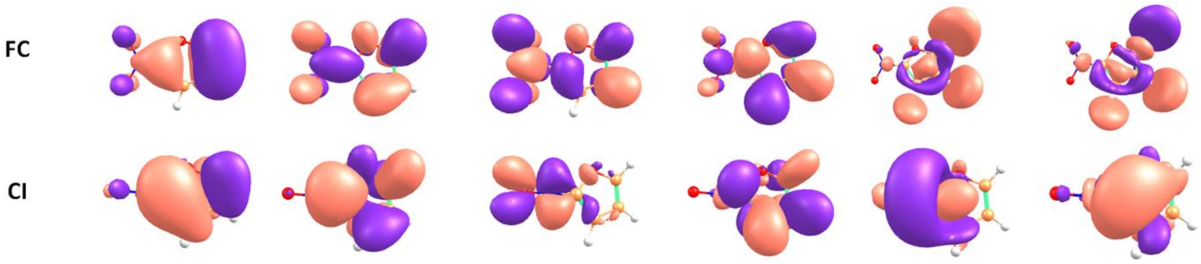

Figure S2: Orbitals included in the active space of the CASSCF calculation in the Franck-Condon point and in the conical intersection shown in Figure 5.

Structures optimized at the B3LYP/aug-cc-pVTZ level (Cartesian coordinates, Ångstrom) along with zero-point corrected energies (Hartree)

C
4H3NO2
E = -434.615507
C 0.808591 1.180159 0.000000
C 2.132440 0.664191 0.000000
C 2.013749 -0.692126 0.000000
O 0.710673 -1.061396 0.000000
C 0.000000 0.086333 0.000000
N -1.424756 -0.018563 0.000000
O -2.042001 1.042652 0.000000
O -1.920865 -1.135470 0.000000
H 0.484639 2.204586 0.000000
H 2.726659 -1.496388 0.000000
H 3.050856 1.224114 0.000000

C4H3NO2–
E = -434.660897
C 1.158696 0.845962 0.000000
C 2.232186 -0.081728 0.000000
C 1.689130 -1.331751 0.000000
O 0.316052 -1.253645 0.000000
C -0.000000 0.082896 0.000000
N -1.316279 0.447271 0.000000
O -1.567751 1.712570 0.000000
O -2.229194 -0.454276 0.000000
H 1.191198 1.918780 0.000000
H 2.109481 -2.338610 0.000000
H 3.306945 0.145591 0.000000

C4H2NO2-
E = -434.036572
C -0.000000 0.054210 -0.000000
C -0.078055 1.430979 -0.000000
C -1.445595 1.718654 -0.000000
C -2.209491 0.526264 -0.000000
O -1.246935 -0.486767 -0.000000
N 1.114939 -0.771578 0.000000
O 0.964580 -2.005636 0.000000
O 2.245929 -0.230050 0.000000
H 0.769729 2.094569 -0.000000
H -1.884049 2.705465 -0.000000

H
E = -0.502260
H 0.000000 0.000000 0.000000

C4H3O-
E = -229.421285
C 0.618966 -1.020007 0.000000
C 1.107214 0.329934 0.000000
C 0.000000 1.116951 0.000000
O -1.122155 0.342078 0.000000
C -0.768351 -1.042685 0.000000
H 1.234572 -1.909187 0.000000
H -0.134837 2.188094 0.000000
H 2.130536 0.679309 0.000000

NO2
E = -205.151718
N 0.000000 0.320841 0.000000
O 1.098243 -0.140390 0.000000
O -1.098243 -0.140346 0.000000

C3OH3-
E = -191.307314
C -0.244369 -1.666964 0.000000
H -1.088640 -2.387517 0.000000
C -0.766209 -0.405271 0.000000
H -1.848102 -0.176414 0.000000
C 0.000000 0.836426 0.000000
H -0.667402 1.741876 0.000000
O 1.208451 1.029613 -0.000000

NO
E = -129.938758
O 0.000000 0.000000 0.534550
N 0.000000 0.000000 -0.610914

CO
E = -113.353802
C 0.000000 0.000000 -0.643326
O 0.000000 0.000000 0.482494

NO2-
E = -205.234330
N 0.000000 0.457495 0.000000
O 1.069389 -0.200196 0.000000
O -1.069389 -0.200112 0.000000

C4H3O
E = -229.353083
C 0.625316 -1.058601 0.000000
C 1.092292 0.306125 0.000000
C 0.000000 1.105765 0.000000
O -1.144312 0.328062 0.000000

C -0.719328 -0.930008 0.000000
H 1.203708 -1.963299 0.000000
H -0.157675 2.167836 0.000000
H 2.118781 0.631282 0.000000

C2H2O-
E = -152.630309
C -1.186881 0.084417 0.000061
C 0.103776 -0.338814 -0.000042
O 1.240764 0.122956 -0.000031
H -1.419442 1.157792 0.000147
H -2.008045 -0.615057 -0.000015

C2HO
E = -151.969923
C 1.255455 -0.130537 0.000021
C -0.020686 0.040774 -0.000040
O -1.191893 0.005912 0.000001
H 2.126533 0.491285 0.000103

C2HO-
E = -152.053511
C 1.253816 -0.115304 -0.000003
C 0.005749 0.024101 0.000027
O -1.214134 0.011612 -0.000030
H 2.155682 0.454322 0.000098

C2H2NO2
E = -282.474641
C 1.930679 -0.048997 0.000029
C 0.800599 -0.687647 -0.000040
N -0.502392 0.046792 0.000004
O -0.480173 1.264389 -0.000030
O -1.501202 -0.651959 0.000036
H 2.327046 0.949592 0.000116
H 0.653032 -1.756714 -0.000126

CH2
E = -39.152415
C -0.000000 -0.000000 0.102763
H -0.000000 0.996110 -0.308290
H -0.000000 -0.996110 -0.308290

C2H3-
E = -77.926298
C -0.067606 -0.776732 0.000000
C -0.067606 0.572915 0.000000
H 0.979743 -1.150917 0.000000
H -0.998264 1.150652 0.000000
H 0.829794 1.223164 0.000000

C2H2-
E = -77.296500
C -0.482881 -0.000181 0.000002
C 0.849357 -0.000166 -0.000001
H -1.100843 -0.918685 -0.000005
H -1.098010 0.920763 -0.000005

COH
E = -113.888439
O 0.061657 -0.589686 0.000000
C 0.061657 0.583074 0.000000
H -0.863201 1.219040 0.000000

C2H-
E = -76.738172
C -0.000000 0.000000 0.489735
C 0.000000 -0.000000 -0.749191
H -0.000000 0.000000 1.556734

COH2
E = -114.525655
O -0.000057 -0.673353 0.000000
C -0.000057 0.527062 0.000000
H 0.938610 1.112445 0.000000
H -0.937806 1.112008 0.000000

CN-
E = -92.890711
N 0.000000 0.000000 0.540638
C 0.000000 0.000000 -0.630744

C3H3O3
E = -341.830255
O 0.544798 -1.929055 0.000000
C -0.624980 -1.532005 0.000000
C -0.975515 -0.142870 0.000000
C 0.000000 0.968918 -0.000000
O -0.382134 2.117289 -0.000000
H -2.015903 0.148688 -0.000000
H -1.448013 -2.259428 0.000000
O 1.297224 0.646424 -0.000000
H 1.387776 -0.330784 0.000000

O-
E = -75.155815
O 0.000000 0.000000 0.000000

C4H3NO2
E = -359.386639
C -0.000000 0.406326 -0.000000
C 1.366807 0.281905 0.000000
C 1.639906 -1.105294 0.000000
C 0.422348 -1.724812 0.000000
O -0.583863 -0.830894 0.000000
N -0.730924 1.579293 -0.000000

O -1.947902 1.461404 -0.000000
H 2.062583 1.102544 0.000000
H 0.133022 -2.761252 0.000000
H 2.600612 -1.589171 0.000000

TS1, Figure 6
E = -434.611528
c 0.228251 0.131398 -0.102501
o -1.346526 -1.299565 0.351592
c -0.646839 1.114591 -0.323066
c -2.038606 0.840195 -0.198353
c -2.292438 -0.469837 0.163014
n 1.565890 -0.029173 -0.016997
o 2.115484 -0.962440 -0.664476
o 2.226336 0.720712 0.764625
h -0.286388 2.093356 -0.633087
h -2.813169 1.567184 -0.387352
h -3.326235 -0.824067 0.290931

LM2, Figure 6
E = -434.622835
c 0.440636 0.166635 -0.224515
o -2.455080 1.243100 -0.250557
c -0.344607 -0.901171 0.133158
c -1.728977 -0.984472 0.238520
c -2.673615 0.060871 0.014104
n 1.802970 0.167406 -0.034484
o 2.344519 1.199245 0.417643
o 2.472431 -0.839333 -0.375547
h 0.208916 -1.816784 0.354191
h -2.152663 -1.946123 0.504100
h -3.732626 -0.284214 0.083176

TS2, Figure 6
E = -434.574832
n 1.809358 -0.023930 -0.105777
c 0.597705 0.269955 0.015040
c -0.405064 1.199504 0.144575
o 1.289293 -1.046660 0.798209
o 2.953946 0.175524 -0.524782
c -1.796869 1.003073 0.142591
c -2.490774 -0.201876 -0.111459
o -1.998944 -1.309910 -0.391895
h -3.598656 -0.099452 -0.063585
h -2.411493 1.875226 0.334999
h -0.039707 2.216165 0.272296

LM3, Figure 6
E = -434.699586
n 2.178381 0.421518 0.120599
c 0.825677 0.467648 -0.239968
c 0.132982 -0.838317 -0.327190
o 0.295801 1.539858 -0.567507
o 2.569176 -0.714549 0.555957
c -1.189739 -1.119641 -0.335775
c -2.331559 -0.291961 0.043046
o -2.352652 0.701668 0.741826
h -3.296351 -0.695953 -0.349192
h -1.487408 -2.117804 -0.652327
h 0.812335 -1.659054 -0.525559

CI, Figure 5
O -1.07992 -0.185261 0.737754
C 0.0707173 -0.531331 0.0318302
C 1.17766 -0.254534 0.873159
C 0.668201 0.115058 2.11082
C -0.705709 0.188972 2.00329
N 0.0115578 0.0386119 -1.32683
O -0.0450681 -0.734008 -2.26242
O 0.0825303 1.24049 -1.48143
H 2.20626 -0.354387 0.589543
H -1.47523 0.156423 2.74615
H 1.22646 0.304346 3.00975
